# Supplementary material for: Association of the Cumulative Live Birth Rate with the Factors in Assisted Reproductive Technology: A Retrospective Study of 16,583 Women
Source: J Clin Med. 2023 Jan 6;12(2):493. doi: 10.3390/jcm12020493 (PMC9862593; doi:10.3390/jcm12020493)
Supplement: Supplementary file 1 [file jcm-12-00493-s001.zip › jcm-2102875-supplementary.pdf]

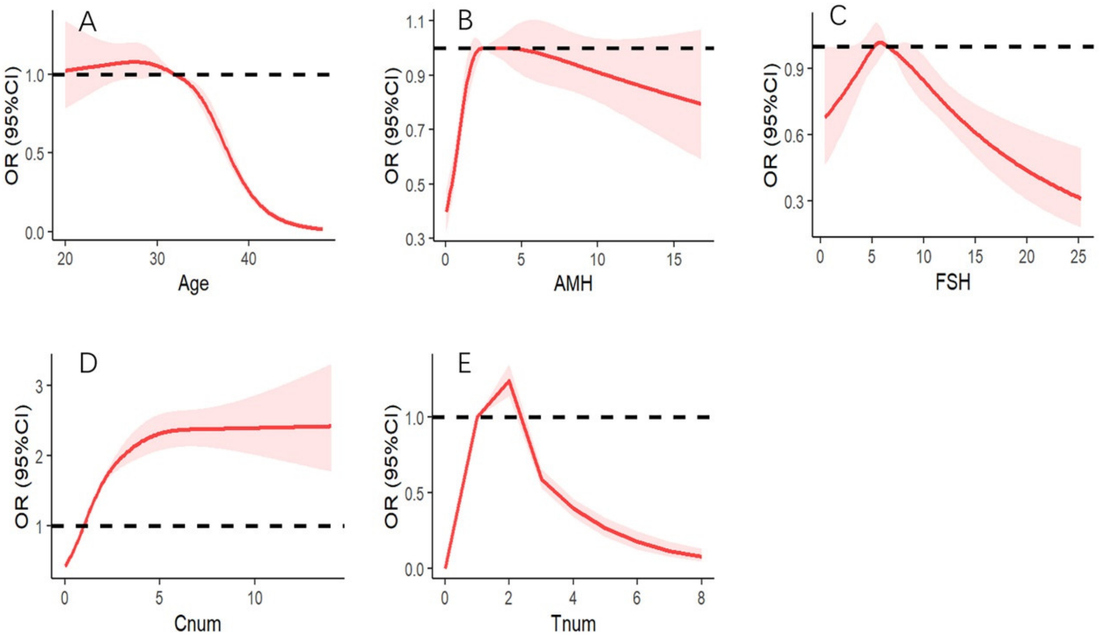

Figure S1. Association of AMH and factors with CLBR in ovulatory women

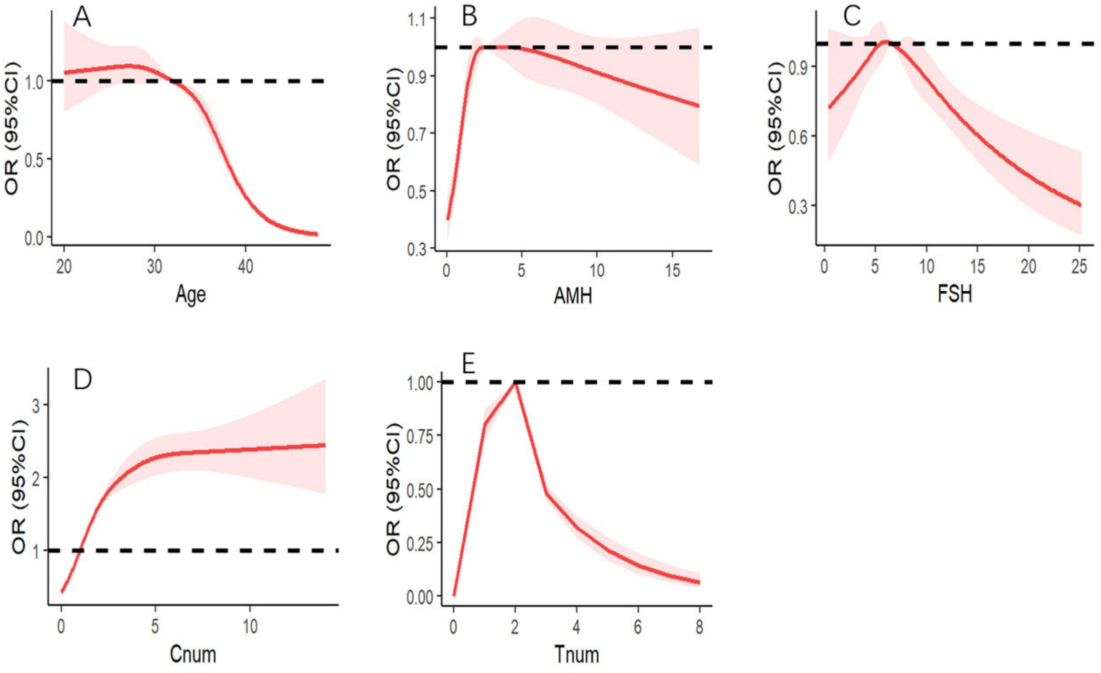

Figure S2. Association of AMH and factors with CLBR in women without endometriosis

AMH, anti-Müllerian hormone; FSH, follicle-stimulating hormone; Cnum, No. of cryopreserved embryos; Tnum, No. of total transferred embryos;
